# Supplementary material for: Are carbon emissions trading and green financial instruments synergistic? -Comprehensive quantitative research based on content analysis
Source: PLoS One. 2024 Mar 7;19(3):e0298601. doi: 10.1371/journal.pone.0298601 (PMC10919601; doi:10.1371/journal.pone.0298601)
Supplement: S1 Data — (ZIP) [file pone.0298601.s001.zip › Data/地级市Z_F工作报告.zip!/╔╜╬≈/┴┘╖┌╩╨/└┤╫╘╣1⁄2╓┌║┼í╛┬φ┐╦╩2╛▌═°í┐_2012.docx]

各位代表：

　　现在，我代表市人民政府，向大会作工作报告，请予审议，并请各位政协委员和其他列席人员提出意见。

　　一、2011年工作回顾

　　2011年，是“十二五”开局之年，也是我市经济社会发展取得重要成果的一年。一年来，在省委、省政府和市委的正确领导下，在市人大、市政协的监督支持下，市政府团结带领全市人民，开拓进取，奋力拼搏，圆满完成了各项预期目标。全年地区生产总值突破千亿大关，达到1135.1亿元，增长15.3%；规模以上工业增加值完成688.亿元，增长23.%；财政总收入完成188.4亿元，增长17.5%；全社会固定资产投资完成645亿元，增长35.1%；城镇居民人均可支配收入达到18924元，增长17.2%；农民人均纯收入达到6084元，增长15.%，主要经济指标均比上年有较大幅度增长，实现了“十二五”良好开局。

　　——转型发展步伐加快。一年来，我们以“转型”为中心，在区域布局上突出“百公里汾河生态城镇经济走廊”和山西国际陆港园区两大重点，大力扶持板块经济。“百公里汾河生态城镇经济走廊”建设迈出重大步伐。成立了管理机构，编制完成了规划方案。汾河城区段生态修复一期工程圆满完成。洪洞赵城煤化工园区、洪洞甘亭工业园区、曲沃优特钢循环工业园区和侯马高新技术电子工业园区建设稳步推进。山西国际陆港园区全面铺开10平方公里的起步区建设，落户企业达28家，成为我市开放型经济发展新亮点。在结构调整上，突出抓循环、抓高端、抓延伸，三次产业结构加速优化。现代高效农业快速发展。粮食总产达21.46亿公斤。建设省级“一村一品”专业村200个，新增设施蔬菜5万亩、水果13.1万亩、中药材3.万亩，肉蛋产量分别增长11.4%和6.6%。吉县苹果、永和红枣、隰县金梨、古县核桃、浮山贡米等品牌效应不断放大；汾西、大宁特色养殖凸显新亮点。尧都尧王台、曲沃磨盘岭、襄汾丁村白莲、洪洞历山、蒲县薛关等现代农业示范园区科技含量、发展规模不断提高。乡宁戎子酒庄、浮山玉杰食用菌等产业化龙头企业不断壮大，实现销售收入达38.2亿元，增长9.1%。传统产业改造和新型产业发展取得重大进展。煤矿企业兼并重组基本完成；尧都、古县、安泽等焦化整合顺利推进；翼城舜达新型锻造基地建设初具规模；山焦60万吨烯烃项目开工建设；山西华翔年产35万吨精密铸件项目竣工投产；飞虹微纳米一期顺利完工。20个市级工业园区实现销售收入880亿元，占全市工业总产值的48%。以旅游为重点的现代服务业健康发展。重点实施了大槐树二期、丁村文化开发等20余项重点旅游景区开发；成功举办了洪洞大槐树祭祖节、安泽荀子文化节等节庆活动，全年旅游综合收入达121.4亿元，增长25％。

　　——城乡面貌明显改观。坚持城乡一体，统筹发展。市区旧城改造和新城建设步伐加快。五一路立交桥主体完工；五一路道路改造、西关桥西延道路一期竣工通车；汾河公园全面建成并向市民开放；公厕建设荣获“中国人居环境范例奖”，在世界厕所峰会上获国际设计大奖；市区框架进一步拉大，功能更趋完善，城镇绿化覆盖率、生活垃圾无害化处理率、集中供热普及率、燃气使用普及率大幅提升。各县(市、区)也不断加大城市基础设施建设力度，乡宁、蒲县、古县、安泽、翼城、洪洞城镇化建设走在了全市的前列。综合交通运输体系建设成效显著。祁临高速临汾北环段、京昆与青兰高速临汾联络线、临吉高速等基本完成，临汾民航机场、大西、中南、张台铁路进展顺利，霍永高速快速推进，新改建县乡公路1018公里。农村新的“五个全覆盖”超额完成年度任务，改造硬化农村街巷13216公里；新建农村便民连锁商店796个；完成农村文化活动室2968个，农家书屋955个，农民休闲健身场所963个；中等职业教育免学费实现全覆盖；新增新型农村社会养老保险试点县7个，总数达到12个。240个新农村建设重点推进村“四化”、“六个一”工程和20个精品示范连片区建设任务全面完成。整村推进87个，移民搬迁5200人。

　　——改革开放深入推进。《资源型经济转型综合配套改革试点行动方案》编制完成，“一市一县”“一市两园”“一县一企”试点工作扎实推进，霍州成功获批国家资源型城市转型试点市，每年可获得国家专项资金支持；乡镇机构改革、医疗卫生体制改革、文化体制改革顺利实施，侯马城乡一体化综合配套改革、安泽新农村建设综合改革取得重大进展；集体林权制度改革，完成勘界确权1048万亩，明晰产权率98.7%；国企改革取得新进展，顺利完成了临纺等五户企业政策性破产。

　　对外开放水平不断提高，参加了“中央企业合作发展恳谈会”、“江浙沪”推介会、第六届中博会等大型招商引资活动，全市共组织广州招商引资推介会等招商活动19次，引进到位外来资金248亿元，签约资金2851亿元，开放型经济迈出新的步伐。

　　——环境保护成效显著。始终把改善环境质量放在政府工作的重要位置，深入开展工业污染治理、城市环境综合整治、农村生态环境保护、水环境治理和蓝天碧水扩容提质五大行动，成功创建省级环保模范城市。新建垃圾处理场10个，建成并投入运营污水处理厂19座。三大水系生态治理取得明显成果。新完成营造林70.万亩。节能减排任务全面完成。全市城区空气质量全部达到国家二级标准，市区达到339天。地表水环境质量持续改善，汾河临汾段主要污染物出境浓度持续下降，市区饮用水质达标率保持100%。洪洞县被评为全国首批资源集约节约模范县。

　　——社会管理全面加强。大力推进平安和谐临汾建设。信访工作全面加强，社会矛盾有效化解，治安状况进一步好转，信访案件上访率和社会治安事件发生率明显下降，全市公众安全感和满意度达到90%以上。不断健全完善突发事件应急处置救援机制，突发事件应急处置水平进一步提高。安全生产“两个主体”责任有效落实，全市安全事故起数和死亡人数分别下降2.8%和8.1%，煤炭百万吨死亡率下降到0.27，安全生产形势持续好转。加强人口和计划生育工作，人口自然增长率控制在4.72‰。全市食品药品、质量监督、科技文化、广播电视、国防人防、民族宗教、外事侨务、地方志、统计、档案、气象、地震、残联、老龄、妇女、儿童、红十字、慈善等各项工作都取得新的进展，为推动临汾经济发展与社会进步作出了积极贡献。

　　——民生状况持续改善。临汾一中高中部投入使用，农村义务教育经费保障机制全面落实，300所标准化学校建设、27所公办幼儿园和5所特教学校新建和改扩建全面完成；高考二本B类以上人数首次突破万人大关，总达线率29.8%。临汾新医院主体完工，市人民医院重新跨入“三甲”行列，28个卫生国债项目顺利推进，基本药物制度全面实行，全市新农合参合率达到97.9%。五大社会保障体系进一步完善，新增城镇就业4.6万人，下岗失业人员再就业1.06万人，转移农村劳动力4.8万人；开工建设保障性住房4540套，完成农村危房改造5700户，落实廉租房租赁补贴562户，907户中低收入家庭喜迁新居。建立物价上涨与社会救助和最低生活保障联动机制，为困难群众发放临时价格补贴5206万元。年初市政府向人民群众承诺的十件实事顺利完成。

　　在加快经济社会发展的同时，市政府坚持把民主法治和自身建设作为做好各项工作的重要保障，自觉接受市人大及其常委会的法律监督和市政协的民主监督，积极支持各民主党派、工商联、无党派人士参政议政。办理人大代表意见建议224件，政协委员提案276件，办结率100%。扎实推进“法治临汾”建设，“六五”普法全面启动。不断完善基层民主制度，第九届村民委员会换届选举工作圆满完成。全面落实党风廉政建设责任制，深入开展机关纪律作风整顿，积极推进政务公开，加强重点行业和领域专项监察和审计监督，政务环境进一步优化，政府的公信力、执行力大幅提升。

　　各位代表，过去的一年，我市继续保持了经济社会平稳较快发展态势，取得了较好成绩。先后荣获“全国文化体制改革工作先进地区”和“五个全覆盖先进市”等表彰113项。这些成绩的取得，是省委、省政府和市委正确领导的结果，是市人大、市政协有效监督和大力支持的结果，更是432万临汾人民团结奋斗、锐意进取的结果。在此，我代表市政府，向全市各条战线的工人、农民、知识分子和广大建设者，向人大代表、政协委员，向各民主党派、工商联、无党派人士和人民团体，向驻临部队、武警官兵、公安政法干警和驻临单位，向所有关心支持临汾发展的社会各界朋友，致以崇高的敬意和衷心的感谢！

　　在充分肯定成绩的同时，我们也清醒地认识到，我市经济社会发展仍然面临不少困难和问题，主要是:产业结构不尽合理，传统产业升级改造步伐还不够快，新兴产业规模还不够大；基础设施、城镇化发展、对外开放和社会管理水平有待进一步提升；安全生产基础仍不牢固，环境保护工作需进一步加强；城乡、区域发展不平衡的矛盾比较突出；保障和改善民生工作与群众的期望还有不少差距；少数部门还存在作风拖拉、办事不力、效能不高等现象。对此，我们将在今后的工作中采取有效措施，认真加以解决。

　　二、2012年工作任务和重点

　　2012年是实施“十二五”规划、加快转型跨越发展的关键之年。当前，面临的外部经济形势依然严峻复杂，国际市场需求萎缩，国内经济增速趋缓，对能源原材料的需求减少，由于我市经济结构的特点，经济增长的压力进一步加大。但总体上看，我市仍然处于加快发展的重大机遇期。国家继续实施中部崛起战略，我省转型综改试验区建设全面推进，为我们提供了良好的发展条件。我们一定要抢抓机遇，乘势而上，坚定信心，扎实工作，团结带领全市人民努力把今年的工作做得更好。

　　今年政府工作的总体思路是：全面贯彻落实党的十七届三中、四中、五中、六中全会、十一届全国人大五次会议、省委十届二次全会暨全省经济工作会议和省十一届人大六次会议精神，以邓小平理论和“三个代表”重要思想为指导，深入贯彻落实科学发展观，按照市委三届二次全会确定的“产业转型、环境提升、城乡统筹、民生改善”的总体要求，以科学发展为主题，以加快转变发展方式为主线，充分运用转型综改试验各项有利政策，牢牢把握“稳中求进、好中求快”的工作基调，突出“主攻项目，加快转型，打造特色，改善民生，推进跨越”的工作重点，进一步优化区域布局、调整经济结构、加强“三农”工作、加快城镇化进程、建设文化强市、保护生态环境、推进改革开放、强化社会管理，不断开创我市经济社会发展新局面，以优异成绩迎接党的十八大胜利召开。

　　“主攻项目，加快转型，打造特色，改善民生，推进跨越”是立足我市市情，全面分析把握当前形势确定的全年工作重点，是今年政府工作的核心。

　　“主攻项目”就是要始终坚持把项目建设作为转型跨越的主要载体和引擎，扎实开展“项目落地年”活动，全面加大项目的投资、协调、服务力度，强力推动项目工作，进一步增强全市经济的发展活力、发展动力和发展后劲。今年重点实施349项重点项目，力争全年完成投资突破1000亿元；项目落地资金达到1500亿元；项目签约达到2000亿元；项目储备保持在12000亿元以上。

　　“加快转型”就是要在发展布局上，以板块经济和园区经济为支撑；在发展路径上，以循环化、高端化、链条化为方向；在增长方式上，更加重视高新技术、高端产品的应用与生产，加快推进传统产业循环化、优势产业规模化、支柱产业多元化、新型产业高端化步伐，努力实现产业结构由煤焦铁等传统产业一枝独大向新型工业、高效农业、现代服务业三次产业协调发展转变，发展模式由粗放、高耗、低效、单一线性发展向集约、低碳、高效、多元循环发展转变，实现绿色发展、清洁发展、安全发展。

　　“打造特色”就是要突出“百公里汾河生态城镇经济走廊”和侯马国际陆港园区两大龙头，打造集新型工业、高效农业、现代城镇、文化旅游、生态文明于一体的经济社会发展引领区和现代物流高地，构建全新的经济形态和社会形态，彰显临汾经济社会发展特色。

　　“改善民生”就是要始终坚持“发展为了人民，发展依靠人民，发展成果由人民共享”的执政理念，把改善民生作为政府工作的出发点和落脚点，紧紧抓住群众关心的、涉及群众切身利益的教育、卫生、社会保障、“三农”工作等热点、难点，实实在在为人民解难题、办好事。

　　“推进跨越”就是要牢牢把握“稳中求进、好中求快”的工作基调，摒弃四平八稳的思维方式，勇于争先，以人一我百、人十我千的精神，坚决超额完成全年各项任务，力争我市经济总量、增长速度、社会管理水平跨入全省先进行列，为圆满实现我市“十二五”翻番目标奠定坚实基础。

　　今年，我市经济社会发展的指导性指标是：地区生产总值增长13%；规模以上工业增加值增长20%；全社会固定资产投资增长25%；社会消费品零售总额增长17%；财政总收入和一般预算收入增长16%；进出口总额增长12%；城镇居民人均可支配收入增长13%；农民人均纯收入增长15%；人口自然增长率控制在6.5‰以内；城镇登记失业率控制在4.2%以内；居民消费价格总水平涨幅控制在4%左右。

　　约束性指标是：万元GDP综合能耗下降3.5%；万元生产总值二氧化碳排放量下降3.7%；二氧化硫、化学需氧量排放量分别下降2%和1.3%；氨氮排放量下降1%；工业烟尘、粉尘排放量下降3%；万元工业增加值用水量下降3%。

　　实现上述目标，我们重点要做好以下十个方面的工作：

（一）继续大力推进“百公里汾河生态城镇经济走廊”建设，努力打造经济发展的优势板块“百公里汾河生态城镇经济走廊”是市委站在历史的高度，从时代发展的要求出发，做出的具有全局性、战略性、引领性的重大决策，符合主体功能区和梯度发展的要求。总体构想是发挥沿汾区域人口集中、区位独特、资源赋存丰富、产业基础较好的优势，在北起霍州，南至侯马，南北长106公里，以汾河为中轴，东西宽约22公里，总面积约2200平方公里的区域内，打造集新型工业、高效农业、现代城镇、文化旅游、生态文明于一体的高度发达的示范区和引领区，带动东西两山全面振兴，形成“沿汾崛起、两翼齐飞”的新格局,成为中西部地区有影响的生态带、产业带、旅游带、城镇带。

　　今年重点完成四项工作：完善规划体系。坚持“统一规划，科学布局，合理分工，统筹推进”的原则，高标准编制完成走廊总体规划以及河道治理、道路建设、城镇建设、新农村建设、生态环保、文化旅游和园区建设等各类专项规划。同时，积极争取国家相关部委及省委、省政府支持，力争把“百公里汾河生态城镇经济走廊”纳入全省先行先试综改试验区规划，上升为全省乃至国家战略。

　　创新体制机制。建立“走廊”内资源统一调配新机制，实现土地、资金、项目、人才等资源的有效利用。建立统一的融资平台，捆绑包装建设项目，加大融资力度，为“走廊”建设提供资金保障。学习借鉴山东、江苏等地在新农村建设、产业园区建设和城镇化等方面的先进经验，加大统筹城乡、统筹区域发展工作力度。创新农村土地流转制度，加快农村土地规模化经营。制定出台新农村改造实施办法，加快农村城市化、城乡一体化步伐。

　　加快基础设施建设。一是全面启动河道水利工程。汛期前完成洪洞至襄汾段河道治理。二是全面启动汾河生态深度治理。

　　完成市区汾河生态治理二期和洪洞城区段滨河生态景观建设。三是建设滨河东路贯通工程。四是启动滨河东路与市级主干道路、各主要产业园区、小城镇连接路及电网配套建设等工程。

　　强化园区支撑。园区经济是现代经济的鲜明特征，也是“走廊”建设的产业支撑和重要内容。按照“主业突出、链条循环、功能完备”的总体要求，全面加快“走廊”内园区建设，突出抓好洪洞赵城煤化工园、洪洞甘亭工业园、尧都河西煤化工园、襄汾绿色铸造科技园、曲沃千万吨级优特钢循环工业园、侯马电子工业园等工业园区，尧都尧王台、洪洞历山、襄汾丁村白莲等现代农业园区，奥特莱斯芭蕾雨、临汾空港等现代物流园区，洪洞广胜寺、霍州七里峪、侯马晋博园等文化旅游景区，把资金、土地等生产要素向园区倾斜和集中，健全服务保障体系，完善园区功能，不断提升园区的项目承载能力、就业吸纳能力和人口容纳能力。

　　各位代表，建设“百公里汾河生态城镇经济走廊”，是我市“十二五”时期的一项重大战略，也是我市转型跨越发展的强大引擎。只要我们全力以赴、坚定不移地下大力气加快推进，就一定能够将“走廊”建设成为山西乃至中西部地区最具竞争力、最有活力的示范区、引领区。

　　（二）进一步加快传统产业新型化和新兴产业规模化步伐，努力打造特色明显的新型工业大市

　　按照以煤为基、以煤兴产、以煤兴业、多元发展的思路，加快推进工业经济结构调整，努力在新型工业化道路上实现新突破。

　　加快煤矿建设步伐。充分发挥煤炭的基础性作用，全力加快煤矿标准化建设。重点针对制约煤矿建设的突出问题，一矿一策，逐矿解决。加强与大企业、大集团的沟通协调，督促帮助其尽快完成建设任务。今年矿井改造完成80%，并建成国家级质量标准化矿井4座，其余生产矿井全部达到省级安全质量标准。同时，在保证安全的前提下，加快煤炭生产，原煤产量达到5300万吨以上。

　　改造升级传统产业。按照“集群化、循环化”的要求，以延长产业链为方向，以循环经济为模式，大力推进传统产业升级改造。焦化行业，抓住整合重组、技术改造和延伸产业链三个环节，积极推进襄汾、洪洞两个焦化生产基地和尧都、古县、安泽、乡宁四个焦化生产集中区建设，大力发展煤制天然气、煤制甲醇、甲醇制烯烃等多联产煤化工。重点抓好山焦60万吨烯烃、山西立恒145万吨焦化、安泽永鑫12万吨甲醇、古县利达20万吨甲醇以及中煤、华电等项目。冶金行业，加快推进“环塔儿山、二峰山”钢铁集群建设，重点抓好中宇钢铁全面复产、翼钢500万吨优特钢项目建设以及襄汾、尧都钢铁产业整合。电力行业，突出发展绿色煤电，促进霍州国电2×60万千瓦发电项目早日投产。同时，抓好安泽冀氏4×60万千瓦、翼城4×60万千瓦坑口发电及古县鑫秀4×60万千瓦煤矸石发电等项目前期工作。

　　培育壮大新兴产业。坚持“规模化、高端化”的原则，把非煤、非资源型产业作为结构调整的战略重点。采用新技术和新工艺，努力在新装备、新能源、新材料、新医药等领域寻求突破。加快发展装备制造业，重点抓好山西华翔精密铸造、平阳重工高端制造、蒲县赢晟园特种铸造、翼城舜达超大型国际一流锻造等项目。大力发展新材料、新技术、新能源，重点抓好尧都光宇电源，洪洞飞虹微纳米LED外延片、芯片及薄膜太阳能电池项目，洪洞恒富美尔美太阳能陶瓷板及外墙保温装饰陶瓷板生产线，吉县、大宁、永和煤层气钻探压缩液化等项目。大力发展生物制药，重点抓好旺龙药业医药产业园等项目。加快科研开发基础建设，在年销售5000万元以上的企业建立研发机构，增强企业科技创新能力；继续资助专利技术申请和项目推广应用，扶持各类科研开发机构加快发展。

　　（三）扎实做好“三农”工作，努力打造现代化新型农村

　　始终把“三农”工作作为重中之重，以农民增收为核心，以“一村一品”“一县一业”为重点，以农业科技创新为支撑，加快农业现代化发展步伐。

　　改善农业生产条件。加强农田水利建设，实施西山沿黄提水灌溉、中线引黄灌溉、禹门口东扩骨干配套、农村饮水安全、病险水库除险加固和中小河流治理等水利工程，全年争取完成投资10亿元以上，完成水土流失初步治理40万亩，实灌面积192万亩。加大农业综合开发力度，力争土地治理总面积达12万亩以上。大力推广机械化保护性耕作、土地深松以及农作物秸秆还田与综合利用，不断提高农业机械化水平。

　　推进农业科技创新。围绕全国“农业科技促进年”活动，进一步完善农业科技服务平台，深入开展农业新技术培训和推广，启动“一乡一站、一村一点”全覆盖工程，大力推广农业科技10大实用技术，培育有文化、懂技术、会经营、善管理的新型农民。组织实施“全民科学素质行动计划纲要”和“百万农民素质提升工程”，完成农民新技术培训100万人次。扎实开展粮食高产创建活动，确保粮食安全。

　　发展现代特色农业。按照“产业特色化、基地规模化、生产标准化、产品品牌化”的要求，发挥优势，突出特色，打造亮点，新增200个省级“一村一品”专业村，进一步做强做大“一县一业”基地县。以建设西山百万亩优质水果基地为重点，促进水果、蔬菜、干果和中药材“四个百万亩”基地规模扩张，全年新增水果10万亩、设施蔬菜3万亩、中药材3万亩、干果9.8万亩。大力发展特色养殖，肉蛋奶产量达到37.5万吨。积极稳妥推进规模经营，大力发展设施农业，建设一批高效农业示范区。今年，每个县市区至少新建一个千亩以上规模较大、标准较高、功能较完善的现代农业园区，带动特色农业、设施农业和精品农业的大发展。

　　培育农业产业化龙头企业。按照“基地化生产、公司化组织、市场化运作、品牌化营销”的思路，重点实施“393”龙头企业培育工程。一方面，加大政策扶持力度，对农业产业化龙头企业在资金、技术、市场等方面给予重点倾斜，今年市政府将拿出专项资金，对重点龙头企业实行贷款贴息支持，引导更多的企业家领办、兼办农产品加工企业。另一方面，整合我市优势农产品资源，加大名优农产品的包装推介和营销力度，扩大品牌知名度和影响力，不断提高农产品加工转化率和市场占有率。同时，大力发展农民专业合作社，培育农村经纪人，提高农民的组织化程度，加快农业产业化进程。

　　加快改善农村面貌。以“四化”和“六个一”工程为重点，大力实施“335”新农村建设推进工程。全面抓好新确定的326个重点村“四化”基础设施建设；高标准建设30个新农村示范连片区和158个农村环境连片整治示范村建设；全面完成4405公里农村街巷硬化，532个农村便民连锁店，690个农家书屋，551个健身场地建设等农村新的“五个全覆盖”任务。

　　扎实推进扶贫开发。以人均收入2300元以下58.6万贫困人口的收入翻番为重点，扎实推进片区开发、移民搬迁、整村推进、产业扶贫、教育扶贫、外资扶贫等项目建设。重点实施大宁——永和特困板块项目，着力打造全省扶贫开发优质果蔬产业示范板块，积极争取将安泽县列为片区开发省级项目县。完成6000人的移民搬迁任务，解决5万人脱贫。

　　各位代表，“三农”工作事关农民切身利益，关乎社会大局稳定。我们要带着对农民兄弟的深厚感情，给农业更强的支持，给农村更大的倾斜，给农民更多的实惠！

　　（四）积极培育物流和旅游两大龙头，努力打造中西部现代服务业大市

　　加快发展以“山西国际陆港园区”为核心的现代物流业。山西国际陆港综合保税园区是发挥方略保税中心物流优势，大力发展外向型经济的前沿区域，也是我市建设中西部物流大市的重要依托。规划面积55平方公里，包括对外贸易集散中心、新兴加工制造业中心、现代物流配送中心，建设陆港物流、工业集群、配套服务三个功能组团，打造成辐射300公里的国际物流陆港、出口加工区和服务业新城，构建晋陕豫黄河金三角商贸物流中心。今年重点推进10平方公里起步区，完成方略卡口、配送中心、交易市场和联检大楼建设，带动我市现代物流业繁荣发展。

　　发展壮大旅游业。按照“一带五区一线十点”的旅游产业发展规划，进一步加快大槐树文化旅游区、尧文化旅游区、中镇霍山旅游区、丁村民俗文化旅游区、晋文化旅游区建设。大力推进洪洞大槐树、吉县壶口瀑布等国家5A级景区创建和洪洞大明风情园、广胜寺整体开发二期、尧陵二期、七里峪景区及克难坡、人祖山等旅游开发项目建设。加大旅游宣传促销力度。举办好大槐树文化节、黄河壶口旅游文化节等旅游节庆活动。积极对接京津客源市场，拓展长三角、珠三角、港澳台客源市场，力争全年旅游总收入达到145亿元，增长20%。

　　做精其他服务业。坚持“大商贸、大市场、大流通”发展战略，扶持发展连锁经营、电子商务，支持优势商贸流通企业做强做大，形成以大型购物中心为骨干，各类超市、专卖店、便利店为支撑，多元化、多层次的城乡商业流通网络。加快发展金融、保险、中介、担保、信息、酒店餐饮等产业，推动各类服务业健康发展。

　　（五）加快推进市域城镇化进程，努力打造百万人口区域性中心城市

　　坚持统筹规划、合理布局、完善功能、以大带小的原则，统筹工业化、城镇化协调共进，加快市域城镇化进程。

　　编制完善城镇发展规划。按照“一带两圈多点”的城镇发展格局，完成涝洰河生态治理、河西新城建设等六个专项规划。加大对各县市区各类专项规划、近期建设规划、控制性详细规划、重要地块修建性详细规划的技术审查力度，发挥好规划引领作用，强化规划的科学性和严肃性。

　　推进“一城三区”同城化发展。按照“唐尧文明圣地、新兴工业之都、和谐宜居新城”的城市定位，大力推进“一城三区”同城化发展。市区按照“建设新城、疏解老城”的方针，加快旧城区道路拓宽改造，启动实施北城壕西段防汛排水及道路建设、古城公园二期、市第一污水处理厂改扩建等工程；完成西赵路西段、中大街北延及北城污水处理厂等工程。新城区着眼完善交通网络，搭建现代化新城框架，启动实施新高中南路、漪汾花园南路、规划十二路、大西客运车站站前广场等工程，完成临汾民航机场、霍侯路改造、桃临线一期、土门高速连接线等交通基础设施建设，加快环保监测大楼、临汾一级客运东站、高铁车站等公共服务设施建设。实施西关村、解放路平阳社区、汾河办盘龙社区等21个城中村、城郊村改造。同步推进洪洞、襄汾同城化建设。

　　不断提升特色大县城和小城镇建设水平。立足县城特色定位，搞好县城规划建设。发挥侯马交通枢纽的区位优势，依托国际物流陆港园区，加快侯马、侯马开发区和曲沃同城化进程。以工矿型、轻工业型和旅游型为主导，推进12个大县城建设、53个重点中心集镇和特色小城镇建设。全市城镇化率提高2个百分点。

　　着力提升城市管理水平。坚持“建管并重”原则，按照“管理内容具体化、管理指标明确化、管理责任区域化”的要求，进一步明确市、区、街道、社区及职能部门职责，有效整合城市管理资源，大力推进城市精细化、标准化管理。以开展“环境建设年”活动为契机，重点开展城市交通秩序、市场经营秩序、建筑和路面污染、广告牌匾规范、环境卫生清理大整治，实现城市规模与管理层次同步提高，城市品位与市民素质同步提升。

　　（六）坚持节能、环保、生态三措并举，努力打造“两型社会”

　　建设资源节约型和环境友好型社会，是经济社会可持续发展的必然要求，也是关系人民群众生活质量和健康安全的重要保障。国家出台环境空气质量监测新标准，对环境保护提出了更高的要求，我们要积极应对，采取更加有力的措施，务求取得更大成效。

　　持续推进节能降耗。全面推进工业、交通、建筑和公共机构节能。坚持能耗强度控制，完善预警调控机制。加强对新建、扩建、改建等固定资产投资项目的能评审查，有效控制能源消费增量。强化技术节能，全面启动“十城万盏”半导体照明应用示范工程。扩大利用风能、太阳能、沼气等可再生能源。

　　切实加强环境保护。巩固省级环保模范城市创建成果，启动“国家级环保模范城市”创建工作。按照新的环境质量监测标准，制定环境保护控制性规划，出台年度行动计划，全力实施建设百公里汾河环保标杆示范区、工业污染治理提效提质、城市环境提质扩容、农村环境连片整治、水环境治理稳中求优五大工程。加快市区及周边工业企业搬迁，治理二次扬尘污染，加强机动车尾气污染防治，优先发展低碳公共交通，加快城中村、城郊村集中供热、供气改造步伐，大力推进天然气、煤层气、焦炉煤气、氢气“四气合一”，建设“气化临汾”，市区大气综合污染指数控制在1.8以下，市区饮用水达标率稳定在100%。

　　全面加快生态建设。深入开展创建“省级园林城市”活动，修编完善《临汾市城市绿地系统规划》，加快中心城区绿化，力争全年城市绿化覆盖率增长1个百分点；大力实施天然林保护、退耕还林、三北防护林、太行山绿化、通道绿化等造林绿化工程，全年完成营造林51.5万亩；继续抓好水土保持、坝滩联治、淤地坝、坡改梯等水保生态工程；重点推进涝洰河生态环境综合治理，努力打造环境优美的生态临汾。

　　各位代表，建设“两型社会”，改善生态环境，既是我们面临的长期任务，也是我们义不容辞的责任。我们要毫不松懈地把这项工作抓实抓好，抓出成效，使我们的天更蓝、地更绿、水更清、空气更清新！

　　（七）推动文化繁荣发展，努力打造三晋文化强市

　　文化是城市的灵魂，是城市综合竞争力的重要体现。我们必须深入贯彻党的十七届六中全会精神，充分发挥我市文化资源优势，坚持“文化强市”之路，推动我市文化大发展大繁荣。

　　努力建设文明临汾。深入开展创建“文明临汾”主题活动，大力弘扬爱国主义、集体主义、社会主义思想和“敢为人先、坚韧不拔、开放包容、勤劳智慧”的临汾精神。加强社会公德、职业道德、家庭美德、个人品德教育，拓展精神文明创建活动，积极推进政务诚信、商务诚信、社会诚信和司法公信，提高全社会文明水平。

　　健全公共文化服务体系。坚持政府主导、社会参与和群众共建共享的原则，加快构建布局合理、功能完善、覆盖城乡的公共文化服务体系。开工建设市广电大楼、图书馆、博物馆、奥体中心等文化重点工程。全面启动县级图书馆、文化馆、体育馆和多功能厅数字影院“三馆一院”建设。继续实施文化惠民工程，实现“农家书屋”、农民体育健身设施、村级文化活动场所“全覆盖”。加快现代传播体系建设，支持网络多媒体、移动多媒体等新兴传播载体快速发展。加强非物质文化遗产保护和传承。广泛开展文化下乡和群众性文体活动，努力满足广大群众的精神文化需求。

　　加快发展文化产业。进一步完善文化产业振兴规划，积极推动文化产业结构调整和转型升级，推进文化与文物、旅游、科技、教育、体育等互动融合。实施文化基础设施提升、文艺精品生产等10大文化产业工程，加快推进侯马晋国古都文化园等15个文化产业园区发展，壮大襄汾锣鼓、邓庄文化用品、翼城花灯、古县“十字绣”等文化产业基地，提升移民文化、丁村文化、帝尧文化、晋国文化、民俗文化、红色文化六大特色文化产业品牌。设立市级文化产业发展专项扶持资金，扶持文化生产经营龙头企业升级改造、做大做强，推动文化产业尽快成为我市新的支柱产业。

　　深化文化体制改革。继续深化公益性文化事业单位内部人事、收入分配、社会保障制度改革，巩固拓展国有文艺演出院团、电影公司、影剧院改革成果。加快经营性文化单位转企改制步伐，鼓励文化企业兼并重组，培植大型演艺、报刊、传媒、网络集团，提高我市文化软实力。

　　（八）深化改革开放，努力打造充满活力的开放临汾

　　全面推进转型综改试验。充分利用好转型综改试验区这个大平台、大载体，在产业转型、生态修复、城乡统筹和民生改善方面先行先试。认真实施《转型综改试验行动方案》，抓好侯马、洪洞、尧都“一市两县”试点工作。推进洪洞赵城煤化工园区、山西国际陆港园区“一市两园”试点建设。加快20个省级试点企业和70个市级标杆项目试点工作，积极探索产业转型升级和促进项目落地建设新途径。同步推进侯马城乡发展一体化综改试点和安泽新农村建设综改试点，带动全市统筹城乡改革扎实推进。

　　进一步深化重点领域改革。理顺国有资产监管体制，解决好临纺等5户政策性破产企业遗留问题，继续稳步推进其他国有企业改革工作。加快行政管理体制和政府机构改革，稳步推进事业单位人事管理制度改革。深化财政管理体制改革，优化支出结构，建设公共财政。全面启动公益林补偿、林权转让和资源评估抵押等林权制度配套改革，基本建立现代林业产权制度。巩固基层医疗卫生体制综合改革成果，启动全市公立医院改革工作。支持银行业改革，推进农村信用社改制为农村商业银行工作，积极引进股份制银行在我市设立分支机构。

　　努力破解发展难题。按照全市“项目落地年”活动的要求，积极推进体制机制创新，力争在项目审批、用地保障、资金筹措等方面取得新突破。加大项目包装和申报力度，争取更多的项目纳入国家和省级投资计划；健全完善领导包项目、部门联项目工作机制，层层落实项目包联责任，建立项目对口推进工作机制，加快项目落地建设。

　　创新土地管理机制，积极争取上级用地指标，加快推进土地开发整理复垦，用足用活城乡建设用地增减挂钩和矿业用地整合政策，力争全年完成造地5.5万亩，力保重点项目和民生工程及时落地。建立多元化融资体系，加强市投资集团、县市区融资平台和信用担保体系建设，整合盘活优质资源，增强融资能力；制定优惠政策，鼓励我市民间资本在本地投资兴业；加快资本市场建设，积极推进企业上市；加强银企合作，采取多种形式，解决发展资金不足的问题。

　　全方位扩大对外开放。以招商引资和招才引智为重点，努力在更宽领域、更高层次参与市场竞争与合作。坚持以资源换资本、换项目、换技术，突出园区招商、产业招商、以商招商，广泛开展各类招商引资活动，努力争取世界500强和国内500强等大型企业来我市投资兴业。积极发展对外贸易，不断提高对外开放水平。研究制定出台引进高端人才、领军人才的优惠政策，继续加大企业、学校、医院等领域高新技术人才的培育和引进力度，为人才扎根临汾创造良好环境。

　　大力发展民营经济。认真落实促进民营经济发展的各项政策，加大对中小企业的扶持力度。鼓励和引导民营企业与大企业、大集团联合，引进项目、技术和资金，转变发展方式，调整产业结构，壮大企业规模。建立健全创业辅导、人才培训、技术支持等中小企业服务平台，切实解决好中小企业贷款担保、人才、技术等发展难题。加快实施企业家素质提升工程，造就一批具有战略眼光、勇于开拓进取的领军人才。大力支持全民创业，激发县域经济发展活力。

　　全年新培育小企业1000户，新增创业基地19个，民营经济总产值、营业收入等主要经济指标增速达到16%以上。

　　（九）以安全生产为重点不断加强和创新社会管理，努力打造“和谐平安临汾”

　　持之以恒抓好安全生产。始终把安全生产作为政府工作的重中之重，牢固树立“抓安全就是抓发展”的理念，进一步强化“两个主体”责任落实，做到职责分工明确、监管不留死角。深入开展安全生产专项整治和打非活动，切实加强煤矿、非煤矿山、尾矿库、危险化学品、道路交通、人员密集场所、消防、特种设备、民爆物品、建筑施工、森林防火、学校、食品药品、校车等各行业、各领域安全生产隐患排查治理。加强安全工作考核，严格实行安全生产“一票否决制”。切实抓好基层基础工作，全面加强安全质量标准化建设，强化安全培训，加强劳动用工管理，突出抓好现场管理，不断创新安全生产监管方式和手段，努力从根本上提高安全生产保障水平。

　　全面加强和创新社会管理。严格落实信访工作责任制，增强责任观念，强化疏堵结合，畅通群众诉求渠道，做好矛盾纠纷排查化解，切实解决好事关人民群众切身利益的突出问题。积极总结推广安泽县“三网合一、三级联动”的做法，夯实社会管理的基层基础。深化平安临汾创建活动，加强社会治安综合治理，完善社会治安防控体系，深入开展打击“两抢一盗”专项行动，严厉打击各类违法犯罪，提高人民群众安全感。

　　进一步加强特殊人员管理，全面推进社区矫正和安置帮扶工作。加强舆情监测，完善互联网舆情应对处置工作机制。健全突发事件应急体系，有效预防和妥善处置各类突发事件。认真落实民族宗教政策，依法管理宗教事务。做好人口与计划生育工作，稳定低生育水平，提高人口素质。广泛开展全民健身活动，提高竞技体育水平。建立健全食盐储备制度，大力推进以绿色低钠盐为主导的食盐健康升级换代工程。加强国防动员和民兵预备役建设，搞好“观摩”活动，推进新形势下的双拥工作。大力发展社会养老服务，完成怡乎贝尔草原山西老年疗养基地怡心家园项目建设。继续做好外事侨务、档案、统计、地方志、妇女儿童、红十字、残疾人、人防、地震、气象等各项工作。

　　（十）更加重视和保障民生，努力打造人民幸福家园优先发展教育事业。加大教育投入，确保财政教育支出占一般预算支出的比例不低于16.96%。继续实施中等职业教育免费全覆盖工程，今年秋季将免费范围扩大到普通中专和技工学校。完成5个县市区薄弱学校改造工程和156所义务教育标准化学校创建任务。继续开展争创“三名”工程活动，确保高考、中考成绩有新的提升。积极发展学前教育，扩大中等职业教育规模。启动山西师大新校区建设、临汾学院整体迁建工程，完成临汾一中高中部二期、初中部教学楼、市第一小学教学楼等工程。

　　大力发展卫生事业。建好临汾新医院、市第九人民医院，开工建设市中医院和市公共卫生应急中心，实施县级卫生监督所建设项目。全面启动市区医疗资源整合工作，进一步优化市区医疗机构布局。强化公共卫生体系和卫生执法体系建设，加强疾病预防控制、社区卫生服务和妇幼保健工作。全面规范医疗机构管理，切实加强医德医风建设。继续做好新农合工作，确保参合率稳定在95%以上。

　　积极实施创业就业。高度关注重点群体、困难群众就业问题，实施更加积极的就业政策，多渠道开发就业岗位。实施好“三支一扶”、就业见习等扶持项目，努力拓展高校毕业生就业渠道。坚持以创业带动就业，深入开展创业培训和职业技能培训，搭建多种形式就业服务平台，促进各类人员就业再就业。

　　全面加强社会保障。进一步扩大社会保险覆盖面，加强社保基金征缴和监管。加快推进城镇居民社会养老保险和新农保工作，确保实现城乡养老保险制度全覆盖。实行新型农村社会养老保险、城镇居民社会养老保险、城镇企业职工养老保险互通互连，实现社会保障“一卡通”。加快经济适用房、廉租房、棚户区改造等保障性住房建设步伐，完善保障性住房分配、运营、退出等管理机制，确保分配公正、管理科学、退出有序。切实加强价格调控和监管，建立健全社会救助和保障标准与物价上涨挂钩的联动机制，切实保障困难群体的基本生活。

　　各位代表，市政府今年将为民办好十件实事：

1.完成临汾民航机场建设工程。

　　2.建成临汾新医院并投入使用。

　　3.新增城市公交车60辆，新开辟市区公交线路5条。

　　4.新建中大街北延等7条城市道路工程。

　　5.新建市图书馆和博物馆。

　　6.新建市奥林匹克中心。

　　7.改善和提高18万农村人口安全饮水标准。

　　8.解决困难家庭就业人员3800人，新增城镇就业47000人。

　　9.全市新建和改扩建公办幼儿园27所。

　　10.新建保障性安居住房15982套。

　　各位代表，民生连着民心，民心凝聚民力。我们一定要不遗余力地把实事办好、好事办实，让人民群众的生活更加幸福美满！

　　三、加强政府自身建设

　　建设美好临汾的新任务，给我们提出了新的更高的要求，我们将紧紧围绕市委三届二次全会暨全市经济工作会议提出的扎实开展“思想解放年、项目落地年、环境建设年、作风转变年”活动的总体要求，牢固树立“以人为本、执政为民”理念，转变政府职能，加强自身建设，提高行政效率，忠实履行服务发展、造福人民的神圣职责。

　　解放思想，建设创新型政府。坚持把解放思想贯穿于经济社会发展的全过程，引导干部群众用世界眼光和战略思维看待事物，推动工作。特别是要紧紧抓住我省“转型综改试验区”有利时机，勇于突破传统思维定式，善于借鉴发达地区成功经验，努力在推动经济发展、加快项目落地、扩大招商引资、加强社会管理、保障改善民生等方面，大胆创新，先行先试，在实践中增强破解发展难题的能力。

　　依法行政，建设法治型政府。自觉接受人大法律监督、工作监督和政协民主监督，认真听取民主党派、工商联、无党派人士和人民团体的意见和建议。健全政府议事规则，坚持依法决策、科学决策、民主决策。严格依照法定权限和程序行使权力、履行职责。加强执法队伍建设，规范行政执法行为。支持人民法院、检察院依法履行职责。

　　全面落实“六五”普法规划，加快法治临汾建设进程。

　　锤炼作风，建设务实型政府。大兴调查研究之风，经常深入基层，深入一线，掌握实际情况，解决实际问题。坚持“干”字当头，“实”字当先，执行力要强、工作节奏要快、方式方法要活、工作效率要高、落实效果要好。对确定的目标不动摇，定下的工作不松手，一项一项地推进，形成加快发展谋大事、心无旁骛干实事、务实扎实干成事的氛围。

　　创优环境，建设高效型政府。牢固树立“政府创优环境、企业创造财富”理念，继续深化行政审批制度改革。清理审批项目，减少审批程序，缩短审批时限，优化审批流程，努力形成零障碍、低成本、高效率的投资环境。对基层、企业、群众反映的问题，要立即办、主动办、认真办、跟踪办。严格落实首问负责制、限时办结制、服务承诺制、责任追究制。加强电子政务建设，大力推行网上审批，为企业、基层、群众提供更加便利、更加高效的服务。

　　勤政廉政，建设廉洁型政府。深入开展保持党的纯洁性学习教育活动，全面贯彻落实党风廉政建设责任制，扎实推进惩治和预防腐败体系建设，着力解决群众反映强烈的突出问题。更加注重勤俭节约，严格控制“三公”经费，加强公务用车管理，进一步降低行政成本。加强对财政资金、重大工程、民生项目的行政监察和责任审计，强化对重点行业、重点领域和重要环节的监督管理。坚决查处各类违法违纪案件，严厉惩治各种腐败行为，努力建设一支清正廉洁的干部队伍。

　　各位代表！时代赋予我们重任，奋斗铸就临汾辉煌。让我们在省委、省政府和市委的坚强领导下，在市人大、市政协的监督支持下，凝心聚力，勇于担当，锐意进取，扎实工作，率先转型，全力跨越，为建设文明开放、富裕和谐新临汾，做出新的更大的贡献，以更加优异的成绩迎接党的十八大胜利召开！
